# Bulk RNA sequencing dataset of hippocampi from RORγt transgenic mice and wild type littermates

**DOI:** 10.1016/j.dib.2026.113361

**Published:** 2026-10-17

**Authors:** Tetsuya Sasaki, Sae Sanaka, Kenyu Nakamura, Suguru Iwata, Momo Morikawa, Yosuke Takei

**Affiliations:** a Laboratory of Anatomy and Neuroscience, Department of Biomedical Sciences, Institute of Medicine, University of Tsukuba, 1-1-1 Tennodai, Tsukuba, 305-8577, Ibaraki, Japan; b College of Medicine, School of Medicine and Health Sciences, University of Tsukuba, 1-1-1 Tennodai, Tsukuba, 305-8577, Ibaraki, Japan; c Master’s Program in Frontier Medical Sciences, Degree Program of Comprehensive Human Sciences, Graduate School of Comprehensive Human Sciences, University of Tsukuba, 1-1-1 Tennodai, Tsukuba, 305-8577, Ibaraki, Japan; d Master’s and Doctoral Programs in Neuroscience, Degree Programs in Comprehensive Human Sciences, Graduate School of Comprehensive Human Sciences, University of Tsukuba, 1-1-1 Tennodai, Tsukuba, 305-8577, Ibaraki, Japan

**Keywords:** Neuroimmune interactions, Rorc, *Mus musculus*, Paired-end sequencing, Gene expression matrix, Data reuse

## Abstract

This dataset comprises bulk RNA-sequencing data from bilateral hippocampi of six untreated, 10-week-old male mice: three heterozygous RORγt-transgenic mice and three wild-type littermates on a C57BL/6 background. Total RNA was extracted using TRIzol, and RNA integrity numbers ranged from 8.1 to 8.6. Ribosomal RNA-depleted, directional libraries were prepared from 500 ng of RNA per sample and sequenced on an Illumina NextSeq 500 with a 2 × 36-bp paired-end configuration. The received reads were 35 or 36 bases long. The 48 original compressed FASTQ files were consolidated into 12 files, preserving 83,357,563 read pairs and their pairing. Original mm10 count and RPKM matrices each contain 49,585 feature entries. An additional GRCm39 reanalysis using Ensembl release 116 provides gene-level fragment counts for 78,348 annotated features, coordinate-sorted alignments and sample-level exploratory analyses. FastQC and MultiQC reports describe raw-read quality, and principal component analysis summarizes variation across all six samples. Original sequencing data and expression matrices are deposited in NCBI Gene Expression Omnibus under accession GSE347403.

Specifications Table

**Table:** 

Subject	Biology
Specific subject area	Hippocampal bulk transcriptomics in a transgenic mouse model
Type of data	Raw paired-end sequencing reads; gene-level count and RPKM matrices; sample metadata and descriptive quality summaries.
Data collection	Bilateral hippocampi from three RORγt-transgenic and three wild-type male mice at 10 weeks. rRNA-depleted, directional libraries; NextSeq 500, 2 × 36-bp configuration (received reads 35–36 bases). Original analysis: CLC 10.1.1/mm10. Revision analysis: Subjunc/featureCounts 2.1.1, GRCm39, Ensembl 116. Tissue collection: 18 July 2018; RNA purification: 19 July 2018.
Data source location	University of Tsukuba, Tsukuba, Ibaraki, Japan. Library preparation and sequencing: Tsukuba i-Laboratory, Transborder Medical Research Center, University of Tsukuba.
Data accessibility	Repository: NCBI GEO. Series accession: GSE347403 [1]. Public release: 16 September 2026. Direct URL: https://www.ncbi.nlm.nih.gov/geo/query/acc.cgi?acc=GSE347403. Raw data: SRA via BioProject PRJNA1529740. The GRCm39 count matrix and annotation are available in GEO under GSE347403 and are also supplied in Supplementary Data 1. Merged FASTQ files, GRCm39 BAM/BAI files and supporting analysis materials have been deposited in Figshare (https://doi.org/10.6084/m9.figshare.34405863).
Related research article	Not applicable (no related research article has been published).

## 1 Value of the Data

• The six individual mouse libraries provide hippocampal expression data for researchers investigating genotype-associated transcriptional variation in this RORγt-transgenic line.
• Original FASTQ files support independent read processing, alignment and quantification using workflows selected for each research question.
• The complete count and RPKM matrices retain low-abundance and zero-valued entries, allowing users to select filters appropriate to their own analytical questions.
• Explicit links between animal identifiers, analysis identifiers, and original filenames facilitate reuse and comparisons with other mouse hippocampal datasets.

## 2 Background

RORγt is a transcription factor involved in the differentiation of IL-17A-producing T helper 17 cells. The Tg(CD2-Rorc)#Staka line expresses murine RORγt under human CD2 regulatory elements and provides a model of sustained alteration of immune regulation [2]. The consequences of IL-17A signaling for neural development and immune–brain interactions depend on developmental stage, tissue context and exposure conditions, as discussed in recent reviews [3,4]. In an experimental maternal immune activation model, Choi et al. showed that maternal RORγt-dependent T cells and IL-17A contributed to cortical abnormalities and altered behavior in offspring [5]. These findings motivate studies of how immune perturbations are associated with brain molecular phenotypes relevant to neurodevelopmental and neuropsychiatric research.

The present study examines untreated adult male RORγt-transgenic mice and wild-type littermates on a C57BL/6 background. This design provides a baseline comparison in a constitutive transgenic line; it does not reproduce an acute maternal immune activation exposure. Previous work in this line reported increased circulating IL-17A, reduced Iba1 immunoreactivity and microglial density in the dentate gyrus, and relatively subtle effects on the hippocampal measures examined [6]. Bulk hippocampal transcriptomes can extend this experimental context by providing a broad inventory of gene-level expression for independent investigation. Because whole hippocampus contains neurons, glia, vascular cells and other cell populations, its expression profile captures both cellular composition and transcription within cells.

This dataset provides paired-end sequencing reads from six individual mice, complete expression matrices and the identifiers needed to connect each library with its biological sample. Researchers can use these resources to formulate hypotheses about immune-related transcription, cellular stress responses and synaptic processes, or to compare the profiles with independently generated mouse brain datasets. Comparisons with human transcriptomic resources require explicit orthologue mapping and attention to species, brain region, age, sex and cell composition. Such comparisons may help prioritize candidate genes or pathways for validation; this small mouse cohort alone cannot establish a mechanism of human disease, an autism-specific signature or a clinical biomarker. The data article focuses on data provenance, quality assessment and reuse. Biological interpretation of differential expression and pathway analyses is addressed in the related research manuscript submitted to Neuropsychopharmacology Reports (Manuscript ID 2766753).

## 3 Data Description

### 3.1 Biological samples and raw data files

Each biological sample represents the bilateral hippocampi of one mouse. WT1–WT3 are wild-type littermates and Tg1–Tg3 are heterozygous transgenic mice. Table 1 links the animal and analytical identifiers to the original FASTQ prefixes. Each prefix is followed by one of L001–L004 and either R1_001.fastq.gz or R2_001.fastq.gz. The four partitions collectively represent one library from one biological replicate.

**Table 1:** Correspondence between biological samples and original sequencing files.

Sample	Analysis ID	Animal ID	FASTQ prefix
WT1	TKS_001_01	402	LAB-160-13_S13
WT2	TKS_001_02	403	LAB-160-14_S14
WT3	TKS_001_03	408	LAB-160-15_S15
Tg1	TKS_001_04	434	LAB-160-16_S16
Tg2	TKS_001_05	435	LAB-160-18_S17
Tg3	TKS_001_06	436	LAB-160-19_S18

For example, LAB-160-18_S17_L001_R1_001.fastq.gz and LAB-160-18_S17_L001_R2_001.fastq.gz form one file pair for Tg2 (animal 435, TKS_001_05). Tg3 corresponds to the LAB-160-19_S18_ prefix. Table 1 preserves the distinct preparation and sequencing identifiers and provides their correspondence. GEO Sample accessions GSM10052262-GSM10052267 correspond, respectively, to WT1, WT2, WT3, Tg1, Tg2, and Tg3.

The original GEO/SRA deposit contains 48 compressed FASTQ files, or 24 R1/R2 file pairs, occupying 5,419,835,409 bytes. For the revision, the four partitions were combined in L001–L004 order separately for R1 and R2, yielding 12 gzip-compressed FASTQ files. No reads were removed or modified. Each merged filename identifies its biological sample, analysis identifier and read direction, for example WT1_TKS_001_01_R1.fastq.gz. A manifest links every original file to its merged file.

### 3.2 Processed expression matrices

Two gzip-compressed tab-separated matrices are available in GEO [1]: GSE347403_TKS_001_gene_counts_mm10.tsv.gz and GSE347403_TKS_001_gene_RPKM_mm10.tsv.gz. Each contains 49,585 data rows and seven columns. Feature_ID preserves the original CLC feature labels; the six remaining columns are TKS_001_01 through TKS_001_06, in the order shown in Table 1. Feature labels are unique and nonblank in these files. Interpretation and conversion to current gene identifiers should use the original annotation as the reference.

The deposited count matrix provides nonnegative integer Total Count values before quantile normalization, alongside the corresponding RPKM abundance matrix. Both matrices retain all 49,585 feature entries, including low-abundance and zero-valued entries, so users can apply filters suited to their analytical questions.

The GRCm39 count matrix contains 78,348 rows identified by Ensembl gene identifiers and six sample columns. Counts are nonnegative integers representing assigned paired-end fragments, and zero-count rows are retained. The accompanying annotation records gene names, biotypes and exon-union lengths. A separate log2(CPM + 1) table supplies the transformed values and a gene-inclusion flag used for PCA; it is an exploratory-analysis input, not a replacement for the integer count matrix.

### 3.3 Recommended use of the files

For reprocessing, use one merged R1/R2 pair per biological sample, or supply the four original partitions per direction together while retaining mate pairing. Table 1 defines the genotype groups and sample identifiers. Choose the original mm10 matrix or the separately generated GRCm39 matrix according to the planned analysis, and document the reference, annotation, count unit and any filtering. The two analyses use different workflows and should not be treated as interchangeable measurements.

### 3.4 RNA quality and sequencing summaries

RNA integrity numbers ranged from 8.1 to 8.6. Table 2 and Fig. 1 report individual read counts from the GRCm39 reanalysis, with 83.4–89.2% mapped. Each original read contributes once; secondary alignments do not add to the denominator. Gene-level quantification instead counts paired-end fragments. The original facility mm10 mapping summary is retained in the accompanying source tables.

**Table 2:** RNA integrity and individual read counts from the GRCm39 reanalysis.

Sample	RIN	Total reads	Mapped reads	Unmapped reads	Mapped (%)
WT1	8.6	30,465,076	25,392,955	5,072,121	83.35
WT2	8.1	26,906,608	23,232,726	3,673,882	86.35
WT3	8.1	29,223,634	25,269,115	3,954,519	86.47
Tg1	8.2	26,213,138	22,765,555	3,447,583	86.85
Tg2	8.2	27,509,560	23,570,180	3,939,380	85.68
Tg3	8.4	26,397,110	23,549,851	2,847,259	89.21

**Fig. 1:**
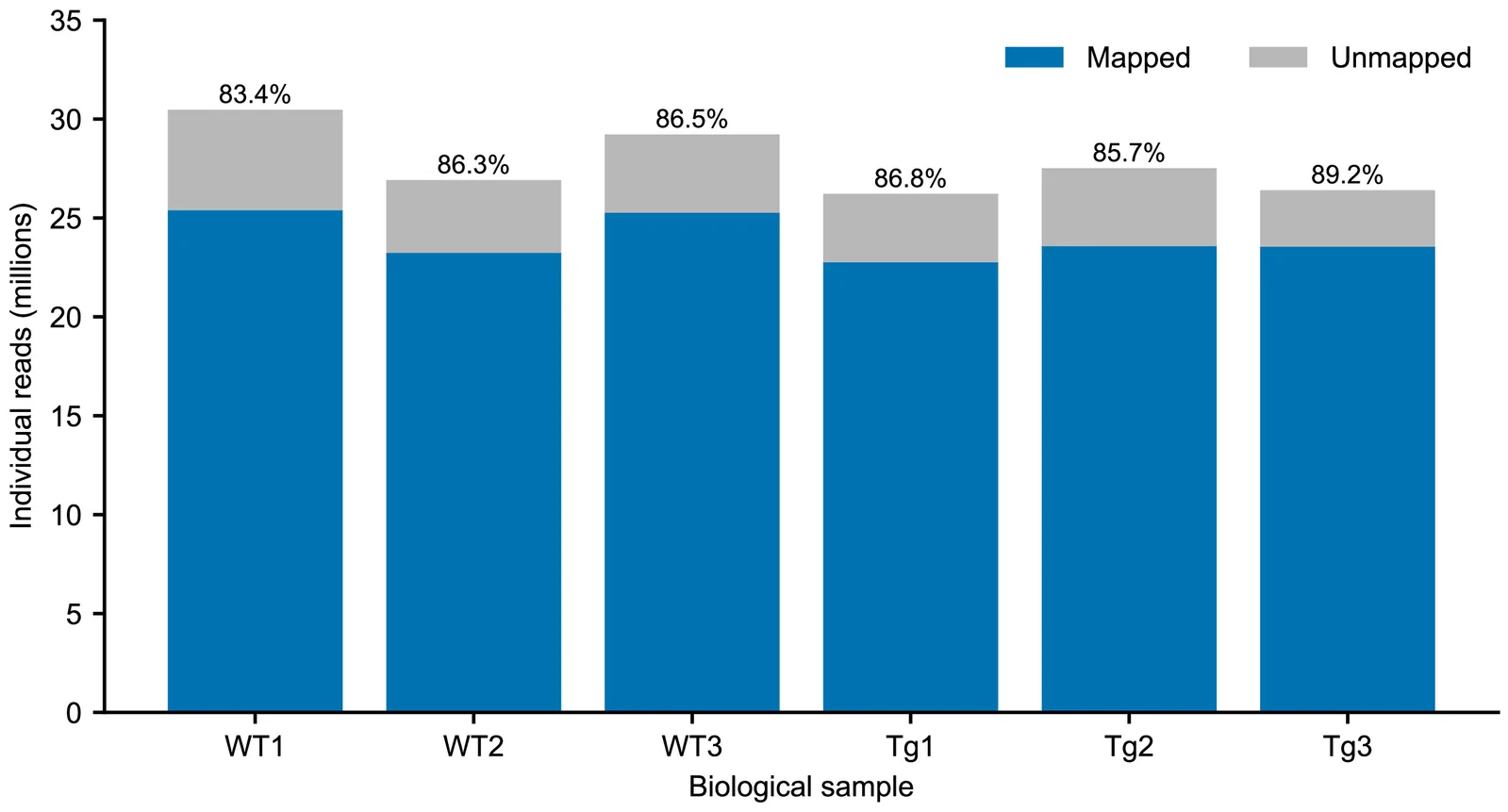
Individual reads mapped to GRCm39. Each stacked bar shows the total individual reads for one biological sample, with primary mapped reads in blue and unmapped reads in gray. Percentages above the bars indicate the mapped fraction. Primary alignments of multimapping reads are included once in the mapped category; gene quantification applies the separate filters described in Methods. Secondary and supplementary alignment records were excluded from this read-level summary. WT, wild type; Tg, RORγt transgenic.

Supplementary Data 1 contains the complete GRCm39 gene-count matrix, gene annotation, transformed expression values and PCA inclusion flags, figure source tables, file-verification manifests, and scripts. Supplementary Data 2 contains individual FastQC reports and the combined MultiQC report. The original mm10 matrices remain available in GEO [1]. The GRCm39 count matrix and annotation are available in GEO under GSE347403 and are also supplied in Supplementary Data 1. Merged FASTQ files, GRCm39 BAM/BAI files and supporting analysis materials have been deposited in Figshare (https://doi.org/10.6084/m9.figshare.34405863). The original GEO/SRA deposit remains available.

All 12 merged FASTQ files passed the FastQC modules for per-base quality, per-sequence quality scores, per-tile quality and adapter content. Mean quality at each read position was at least 30.78 on the Phred scale (Fig. 2). All files contained both 35- and 36-base reads. FastQC flagged per-base sequence composition in all files, and reported warnings for GC distribution, N content, variable read length and overrepresented sequences. Sequence duplication estimates ranged from 45.45% to 57.48%, producing eight warnings and four failures. These sequence-level duplication estimates do not distinguish PCR duplicates from repeatedly sampled abundant transcripts. The reports retain every module and its status for reader assessment.

**Fig. 2:**
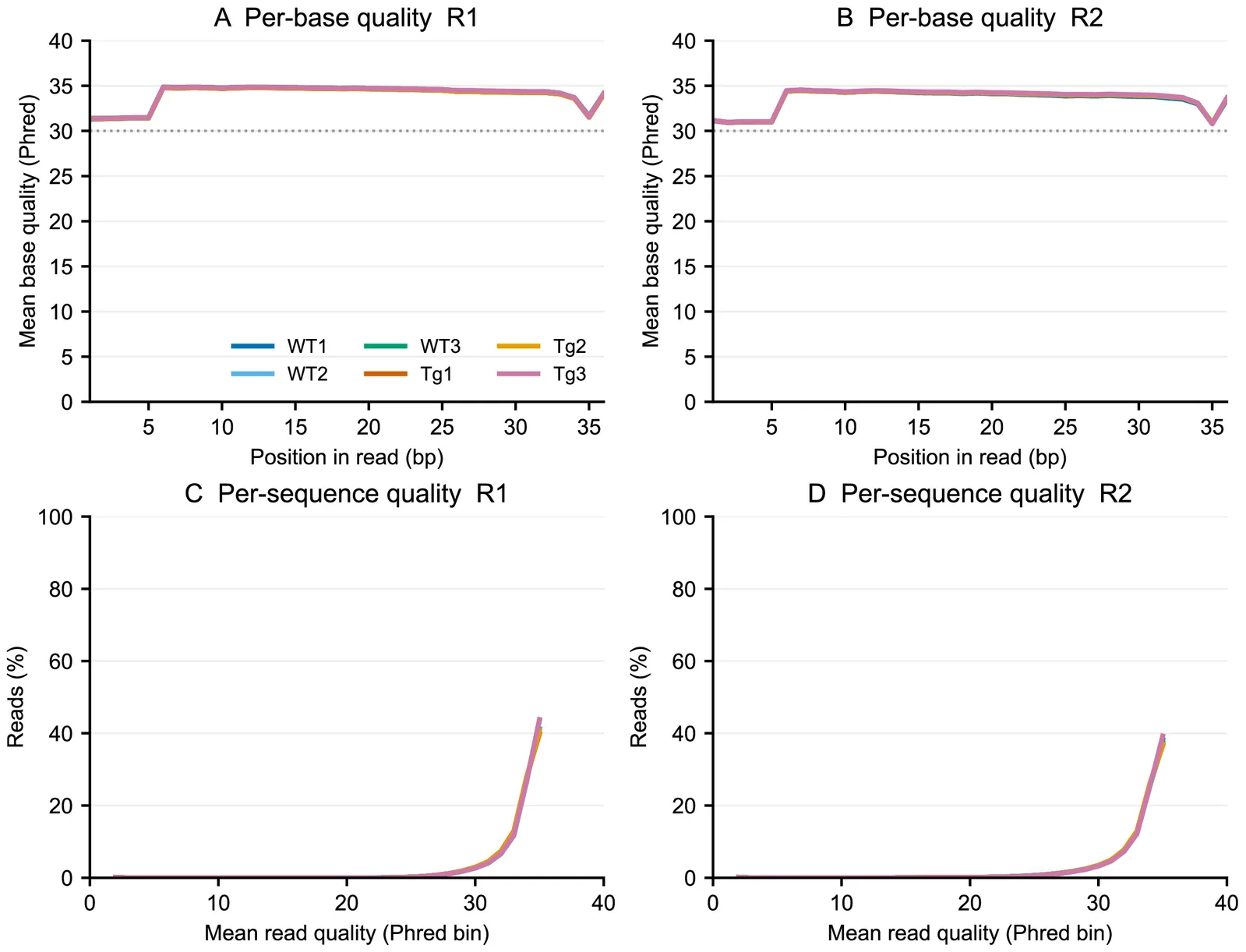
Quality profiles of the merged raw reads. Panels A and B show mean per-base Phred scores for R1 and R2, respectively; the dotted line marks Phred 30. Panels C and D show the distribution of mean per-read quality scores, expressed as a percentage of reads in each file. All six biological samples are shown with consistent colors. Full FastQC distributions, module statuses and MultiQC summaries accompany the figure.

PCA used 17,689 genes that met the expression filter (Fig. 3). PC1 and PC2 explained 26.0% and 20.7% of the variance, respectively. The first two components did not show consistent separation of the three wild-type and three transgenic samples. WT3 was close to Tg2 and Tg3 in this projection, while WT1, WT2 and Tg1 occupied distinct positions. Pairwise Pearson correlations across the same transformed genes were high (0.9885–0.9910), including comparisons between genotypes. These observations describe variation within this small cohort; high overall correlations do not establish a genotype effect or rule out technical or biological confounding. All six samples were retained.

**Fig. 3:**
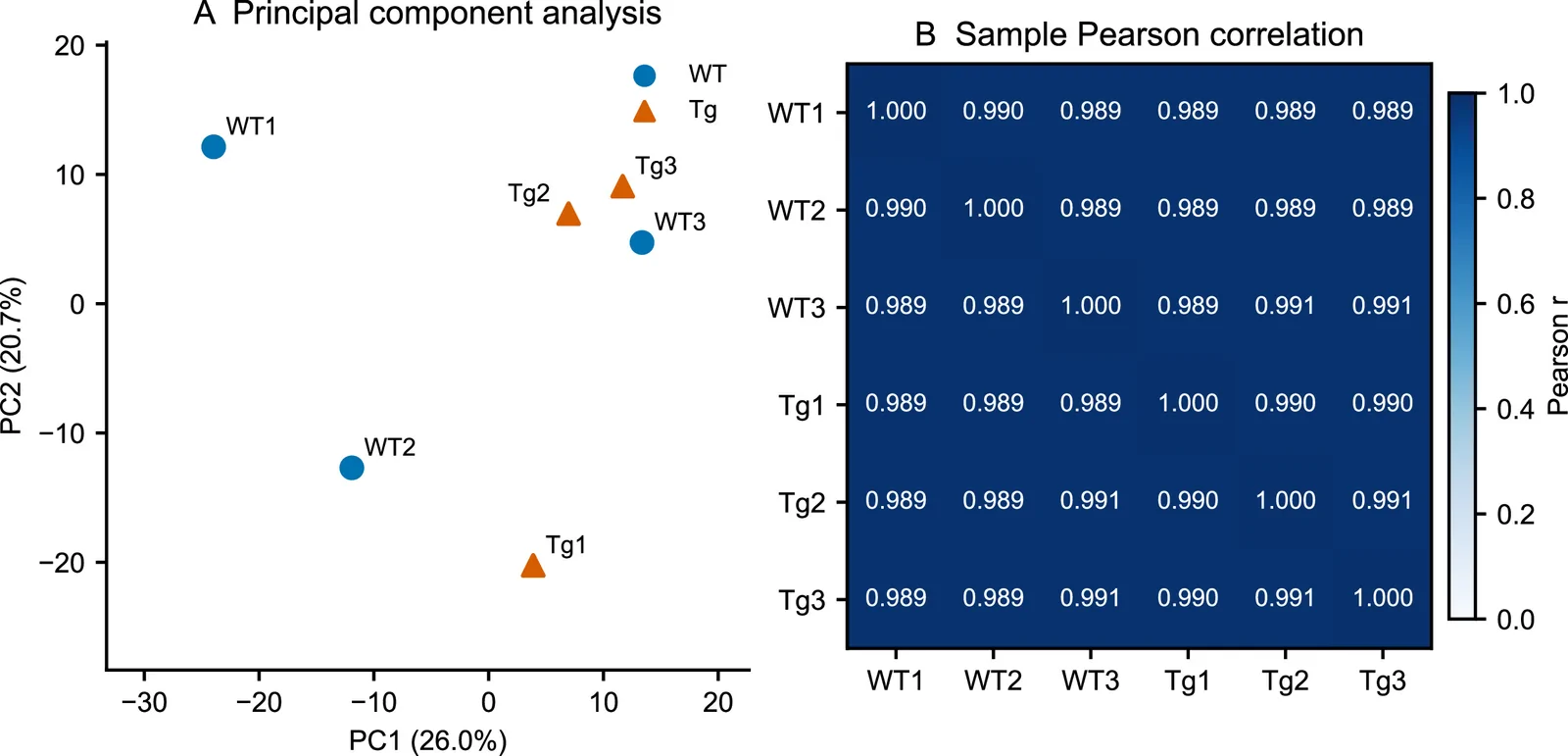
Exploratory analysis of GRCm39 gene counts. (A) PCA of log2(CPM + 1) values for 17,689 genes with CPM ≥ 1 in at least three samples. Expression values were centered per gene across all six samples without variance scaling. Axis labels show explained variance. Each point is one mouse; every sample is labeled and retained. (B) Pearson correlations between samples using the same transformed, filtered matrix. These plots describe sample relationships and do not constitute a statistical test of genotype effects.

## 4 Experimental Design, Materials and Methods

### 4.1 Animals and tissue collection

Heterozygous Tg(CD2-Rorc)#Staka mice (MGI:5805451) were maintained on a C57BL/6 background by backcrossing, consistent with the previously described maintenance of this line [7]. Three male transgenic mice and three male wild-type littermates were sampled at 10 weeks of age on 18 July 2018. Animals were maintained under specific-pathogen-free conditions at the Laboratory Animal Resource Center, University of Tsukuba, with a 12-h light/12-h dark cycle and unrestricted access to food and water. Samples were collected under baseline conditions. Animals were euthanized by cervical dislocation followed by decapitation. Tissue collection was performed between 14:00 and 17:00. Both hippocampi were dissected and immersed in RNAlater Stabilization Solution (Invitrogen/Thermo Fisher Scientific, AM7020) within 30 min of decapitation. Samples were held at 4 °C overnight before RNA purification on 19 July 2018. Samples were selected for RNA sequencing on the basis of the condition of the dissected hippocampal tissue. Randomization and blinding were not performed.

### 4.2 Tail DNA extraction and genotyping

Genomic DNA was extracted from approximately 5-mm tail biopsies by alkaline lysis in 600 µL of 50 mM NaOH at 95 °C for 30–120 min until tissue disintegration. Lysates were neutralized with 50 µL of 1 M Tris–HCl (pH 8.0) and centrifuged at 15,000 rpm for 5 min at 4 °C. The supernatant was used as the PCR template at 5% of the total reaction volume.

Genotyping PCR was performed using BIOTAQ DNA polymerase for 35 cycles with an annealing temperature of 57 °C and an extension time of 45 s. The forward primer was 5′-CATCATCTCTGCAAGACTCA-3′ and the reverse primer was 5′-TGAGTTCGAAGATGCAGAGG-3′, with an expected transgene amplicon size of 667 bp. Genotype assignments recorded in the mouse management notebook were consistent with the RNA-sequencing sample identifiers: animals 402, 403 and 408 were wild type, and animals 434, 435 and 436 were transgenic.

### 4.3 RNA extraction and library preparation

Total RNA was extracted with TRIzol reagent (Thermo Fisher Scientific/Invitrogen, 15596026) and resuspended in water. RNA was evaluated using a NanoDrop spectrophotometer and an Agilent Bioanalyzer with the RNA 6000 Pico Kit (5067–1513). Individual RIN values are provided in Table 2. Library preparation and sequencing were performed by Tsukuba i-Laboratory, Transborder Medical Research Center, University of Tsukuba.

The facility confirmed that the library reagents listed below were used in 2018. For each sample, 500 ng of total RNA underwent ribosomal RNA depletion with the NEBNext rRNA Depletion Kit (Human/Mouse/Rat; New England Biolabs, E6310), followed by directional library preparation with the NEBNext Ultra Directional RNA Library Prep Kit for Illumina (E7420). The facility workflow specified library assessment using the Agilent Bioanalyzer High Sensitivity DNA Kit (5067–4626). Sequencing used an Illumina NextSeq 500 with a High Output Kit v2 and 2 × 36-bp paired-end reads.

### 4.4 Original mm10 alignment and gene quantification

FASTQ partitions were concatenated separately for R1 and R2 and imported as paired-end reads into CLC Genomics Workbench version 10.1.1 (QIAGEN). Reads were mapped to the mouse mm10 reference genome and quantified for 49,585 gene-level features using Ensembl-derived gene and transcript annotations distributed with the software. Total counts were normalized using the quantile method, and differential gene expression was analyzed using the Empirical Analysis of DGE tool in CLC Genomics Workbench. The complete unnormalized Total Count and RPKM matrices were retained from the original output for the GEO deposit. The deposited matrices preserve the complete original gene-level quantification before differential-expression filtering.

### 4.5 Raw file integrity

Fresh MD5 checksums for all 48 original compressed FASTQ files matched both the provider manifest and the historical verification record. All FASTQ records were checked for four-line structure, matching sequence and quality lengths, and valid quality characters. R1/R2 read identifiers and record counts agreed in each of the 24 original partition pairs. After merging in partition order, every output was decompressed in full and its SHA-256 digest was compared with the concatenated original uncompressed stream. All 12 outputs agreed exactly, preserving 166,715,126 individual reads (83,357,563 read pairs). MD5 checksums were also recorded for the merged compressed files.

### 4.6 Processed data consistency

The six count columns and six RPKM columns in the complete 49,585-row intermediate table were compared with the original facility workbook. All 595,020 numeric values agreed. The two deposited matrices were then compared with the intermediate table by feature label and sample, with relative and absolute numeric tolerances of 10⁻¹². All compared values agreed within these tolerances, all values were finite and nonnegative, and every count value was an integer.

### 4.7 GRCm39 alignment and fragment quantification

For the revision, merged reads were aligned without additional trimming or filtering to the GRCm39 primary assembly (mm39), using Subjunc 2.1.1 [8] and the matching Ensembl release 116 GTF. Reference and annotation files were retrieved from the versioned Ensembl distribution; gzip integrity and the provider checksums were verified. A gapped Subread index was built with a 6000-MB memory target. Alignment used 14 subreads, anchor and non-anchor consensus thresholds of one, at most three mismatches and five-base indels, Phred+33 encoding, forward/reverse mate orientation and fragment-length parameters of 50–600 bases. Multimapping reads were reported with up to ten equally best alignments. BAM files were sorted by genomic coordinate and indexed. Exact commands, software versions, reference URLs and file checksums are supplied with the analysis scripts.

Read-level summaries and gene quantification used a temporary BAM containing one primary record per original read. Secondary and supplementary records were excluded using SAM FLAG mask 0x900. Subjunc reported repeated unmapped-mate records for some alternative alignments. Records sharing a read name and mate direction were audited for identical original-orientation sequence and quality, and the unmapped record with hit index HI = 1 was retained to correspond to the mapped primary mate. Mapped primary records were unique. Total records, R1/R2 totals and complete-pair counts were checked against FASTQ totals for each sample. Full-BAM Picard CollectAlignmentSummaryMetrics 3.5.0 totals were reconciled with the repeated unmapped records. This procedure consolidates alignment records; it does not remove PCR or biological duplicates. The full BAM files generated for this reanalysis retain all reported alignments.

Gene-level fragments were counted with featureCounts 2.1.1 [9], aggregating exon features by gene_id. The temporary BAM retained the original NH tags, allowing multimapping fragments to be excluded. Both mates were required to be mapped; chimeric pairs and fragments overlapping multiple genes were excluded. At least one mate had to meet mapping quality 10. Reverse-stranded counting was used for the directional libraries, supported by a comparison of unstranded, forward-stranded and reverse-stranded assignment in WT1. Each original fragment was accounted for in exactly one featureCounts summary category, and each count-column sum equalled its Assigned total. Full annotation rows, including zero-count genes, were retained.

### 4.8 Read quality and exploratory sample analysis

FastQC 0.13.0 (Babraham Bioinformatics, https://www.bioinformatics.babraham.ac.uk/projects/fastqc/) was run on each of the 12 merged FASTQ files. MultiQC 1.35 [10] combined the reports. Plotting used the numeric FastQC module outputs without removing warning or failure modules. For PCA, gene counts were converted to counts per million using the total assigned gene fragments in each sample and transformed as log2(CPM + 1). Genes with CPM ≥ 1 in at least three of six samples were retained using a filter independent of genotype labels. PCA was calculated by singular value decomposition after centring each gene across samples, without scaling genes to unit variance. Pearson sample correlations used the same matrix. All six samples were retained; no filtering based on group separation was performed.

## Limitations

The dataset provides three biological replicates per genotype from male mice sampled at one reported age, supporting exploratory comparisons within this cohort. This sample size limits statistical power and the precision of estimates of biological variability. Generalization across sexes and ages requires additional cohorts. Bilateral whole-hippocampus measurements integrate multiple regions and cell populations; resolving cellular sources and separating cell-composition effects from within-cell expression changes requires complementary measurements. Findings in this constitutive transgenic model reflect the combined context of hippocampal, systemic and developmental influences. Samples were selected by dissected tissue condition, and randomization and blinding were not performed. Sample-level clustering describes the observed expression structure; it does not independently establish biological validity, and genotype separation is not a prerequisite for retaining a sample.

Sequencing was configured for 2 × 36-bp paired-end reads. Short paired-end reads can support gene-level quantification for well-annotated genes, but provide limited sequence information for novel transcript assembly, complex alternative splicing and isoform-level inference. Repeated or highly similar genomic regions can produce ambiguous alignments, reducing unique mapping and limiting the interpretation of homologous genes. A current reference assembly improves the reference resource available for analysis but does not remove these read-length constraints. The received files contained 35- and 36-base reads. Sequence-composition and duplication flags also warrant consideration when selecting a reuse workflow; high sequence duplication by itself does not establish its biological or technical origin.

The original mm10 analysis used CLC Genomics Workbench 10.1.1, quantile normalization and the Empirical Analysis of DGE tool. Exact reproduction requires additional details of the original annotation release and preprocessing, mapping, counting and differential-expression settings. The original count and RPKM matrices preserve that analysis. The GRCm39 reanalysis uses a separately documented workflow; differences between the outputs may reflect the aligner, annotation and counting rules as well as the reference assembly.

## Ethics Statement

Animal procedures were conducted in accordance with University of Tsukuba animal-care guidelines and the National Institutes of Health Guide for the Care and Use of Laboratory Animals. The study was approved by the Animal Experiment Committee of the University of Tsukuba (approval numbers 18–148 and 19–150). Recombinant DNA experiments were approved by the University of Tsukuba under approval number 170021. The dataset comprises six male animals.

## CRediT Author Statement

**Tetsuya Sasaki:** Conceptualization, Methodology, Validation, Formal analysis, Investigation, Resources, Data curation, Writing – original draft, Writing – review & editing, Visualization, Supervision, Project administration, Funding acquisition. **Sae Sanaka:** Validation, Investigation, Data curation, Writing – review & editing. **Kenyu Nakamura:** Methodology, Validation, Formal analysis, Investigation, Data curation, Visualization, Writing – review & editing. **Suguru Iwata:** Resources, Writing – review & editing. **Momo Morikawa:** Resources, Writing – review & editing. **Yosuke Takei:** Conceptualization, Resources, Supervision, Project administration, Writing – review & editing.

## Declaration of generative AI and AI-assisted technologies in the manuscript preparation process

During preparation of this work, ChatGPT (OpenAI) assisted with English drafting, data-table checking and development of scripts for file-integrity verification, the reanalysis workflow, quality-report processing and scientific plotting. The sequencing reads and measurements were derived from the experimental files; no synthetic experimental data or experimental images were generated.

## Data Availability

GEOHippocampal interferon-response, unfolded-protein-response and synaptic transcriptional signatures in RORgt-transgenic mice (Original data)

## Appendix Supplementary materials

Image, application 1

Image, application 2
