# Supplementary material for: Bulk RNA sequencing dataset of hippocampi from RORγt transgenic mice and wild type littermates
Source: Data Brief. 2026 Oct 17;69:113361. doi: 10.1016/j.dib.2026.113361 (PMC13743875; doi:10.1016/j.dib.2026.113361)
Supplement: Supplementary file 2 [file mmc2.zip › Supplementary_Data_2_FastQC_MultiQC/fastqc/Tg1_TKS_001_04_R1_fastqc.html]

Tg1\_TKS\_001\_04\_R1.fastq.gz FastQC Report 

FastQC Report

�� 12 10�� 2026  
Tg1\_TKS\_001\_04\_R1.fastq.gz

## Summary

- Basic Statistics
- Per base sequence quality
- Per tile sequence quality
- Per sequence quality scores
- Per base sequence content
- Per sequence GC content
- Per base N content
- Sequence Length Distribution
- Sequence Duplication Levels
- Overrepresented sequences
- Adapter Content

## Basic Statistics

| Measure | Value |
| --- | --- |
| Filename | Tg1\_TKS\_001\_04\_R1.fastq.gz |
| File type | Conventional base calls |
| Encoding | Sanger / Illumina 1.9 |
| Total Sequences | 13106569 |
| Total Bases | 468.1 Mbp |
| Sequence length | 35-36 |
| Mean Length | 35 |
| Median Length | 36 |
| %GC | 56 |

## Per base sequence quality

## Per tile sequence quality

## Per sequence quality scores

## Per base sequence content

## Per sequence GC content

## Per base N content

## Sequence Length Distribution

## Sequence Duplication Levels

## Overrepresented sequences

| Sequence | Count | Percentage | Possible Source |
| --- | --- | --- | --- |
| CGGGTATCTGGCTTCCTCGGCCCCGGGATTCGGCG | 73872 | 0.56 | No Hit |
| CTGGCTTCCTCGGCCCCGGGATTCGGCGAAAGCTGC | 71468 | 0.55 | No Hit |
| GTCCAGAGTCGCCGCCGCCGCCGGCCCCCCCGAGTG | 63943 | 0.49 | No Hit |
| CTCGGCTTCGTCGGGAGACGCGTGACCGACGGTCCC | 61457 | 0.47 | No Hit |
| GTCGGCTGCTAGGCGCCGGCCGAGGCGAGGCGCCGC | 45403 | 0.35 | No Hit |
| CCCGAGTGTCCGGGCCCCCCGCCCCACCGGGGGCCC | 42380 | 0.32 | No Hit |
| CTGGAGTCTTGGAAGCTTGACTACCCTACGTTCTCC | 33317 | 0.25 | No Hit |
| GTCGCGGCGTAGCGTCCGCGGGGCCCGACGCCGCGG | 30111 | 0.23 | No Hit |
| CTCCTACTCGTCGCGGCGTAGCGTCCGCGGGGCCCG | 29470 | 0.22 | No Hit |
| GGGTAGCCGACATCGCCGCCGACCCCGTGCGCTCGG | 27488 | 0.21 | No Hit |
| CGGGTGGGTAGCCGACATCGCCGCCGACCCCGTGCG | 25864 | 0.2 | No Hit |
| GTTCAACGTGACGGGCGAGAGGGCGGCCCCCTTTCC | 25610 | 0.2 | No Hit |
| ATCTGGCTTCCTCGGCCCCGGGATTCGGCGAAAGCT | 22334 | 0.17 | No Hit |
| CCAGGCTGGAGTGCAGTGGCTATTCACAGGCGCGAT | 21857 | 0.17 | No Hit |
| CTCCGTTTCCGACCTGGGCCGGTTCACCCCTCCTT | 21448 | 0.16 | No Hit |
| CTCGCTATGTTGCCCAGGCTGGAGTGCAGTGGCTAT | 19998 | 0.15 | No Hit |
| CCCGTGCGCTCGGCTTCGTCGGGAGACGCGTGACCG | 18513 | 0.14 | No Hit |
| GGGGAAGAGAGGTGGCGACGACGCGGGGGACGACGG | 18208 | 0.14 | No Hit |
| CGCGGCGTAGCGTCCGCGGGGCCCGACGCCGCGGGG | 17984 | 0.14 | No Hit |
| CCCTCCTTAGGCAACCTGGTGGTCCCCCGCTCCCGG | 17530 | 0.13 | No Hit |
| CCAGAGTCGCCGCCGCCGCCGGCCCCCCCGAGTGTC | 17393 | 0.13 | No Hit |
| GCCGACATCGCCGCCGACCCCGTGCGCTCGGCTTCG | 17088 | 0.13 | No Hit |
| CTCGCGTCCAGAGTCGCCGCCGCCGCCGGCCCCCCC | 16159 | 0.12 | No Hit |
| GTCCGCGGGGCCCGACGCCGCGGGGGCGAAACCCGG | 15946 | 0.12 | No Hit |
| CCCCGAGTGTCCGGGCCCCCCGCCCCACCGGGGGCC | 14274 | 0.11 | No Hit |
| CCCCTCCTTAGGCAACCTGGTGGTCCCCCGCTCCCG | 14065 | 0.11 | No Hit |
| CGGCTGCTAGGCGCCGGCCGAGGCGAGGCGCCGCGC | 13704 | 0.1 | No Hit |
| CTCGTCGCGGCGTAGCGTCCGCGGGGCCCGACGCCG | 13122 | 0.1 | No Hit |

## Adapter Content

Produced by FastQC (version 0.13.0)
