# Supplementary material for: Bulk RNA sequencing dataset of hippocampi from RORγt transgenic mice and wild type littermates
Source: Data Brief. 2026 Oct 17;69:113361. doi: 10.1016/j.dib.2026.113361 (PMC13743875; doi:10.1016/j.dib.2026.113361)
Supplement: Supplementary file 2 [file mmc2.zip › Supplementary_Data_2_FastQC_MultiQC/fastqc/Tg2_TKS_001_05_R1_fastqc.html]

Tg2\_TKS\_001\_05\_R1.fastq.gz FastQC Report 

FastQC Report

�� 12 10�� 2026  
Tg2\_TKS\_001\_05\_R1.fastq.gz

## Summary

- Basic Statistics
- Per base sequence quality
- Per tile sequence quality
- Per sequence quality scores
- Per base sequence content
- Per sequence GC content
- Per base N content
- Sequence Length Distribution
- Sequence Duplication Levels
- Overrepresented sequences
- Adapter Content

## Basic Statistics

| Measure | Value |
| --- | --- |
| Filename | Tg2\_TKS\_001\_05\_R1.fastq.gz |
| File type | Conventional base calls |
| Encoding | Sanger / Illumina 1.9 |
| Total Sequences | 13754780 |
| Total Bases | 491.3 Mbp |
| Sequence length | 35-36 |
| Mean Length | 35 |
| Median Length | 36 |
| %GC | 56 |

## Per base sequence quality

## Per tile sequence quality

## Per sequence quality scores

## Per base sequence content

## Per sequence GC content

## Per base N content

## Sequence Length Distribution

## Sequence Duplication Levels

## Overrepresented sequences

| Sequence | Count | Percentage | Possible Source |
| --- | --- | --- | --- |
| CTCGGCTTCGTCGGGAGACGCGTGACCGACGGTCCC | 91263 | 0.66 | No Hit |
| CGGGTATCTGGCTTCCTCGGCCCCGGGATTCGGCG | 80884 | 0.59 | No Hit |
| CTGGCTTCCTCGGCCCCGGGATTCGGCGAAAGCTGC | 74245 | 0.54 | No Hit |
| GTCCAGAGTCGCCGCCGCCGCCGGCCCCCCCGAGTG | 49891 | 0.36 | No Hit |
| CCCGAGTGTCCGGGCCCCCCGCCCCACCGGGGGCCC | 46046 | 0.33 | No Hit |
| GTCGCGGCGTAGCGTCCGCGGGGCCCGACGCCGCGG | 43422 | 0.32 | No Hit |
| CTCCTACTCGTCGCGGCGTAGCGTCCGCGGGGCCCG | 40777 | 0.3 | No Hit |
| CTGGAGTCTTGGAAGCTTGACTACCCTACGTTCTCC | 36813 | 0.27 | No Hit |
| GTCGGCTGCTAGGCGCCGGCCGAGGCGAGGCGCCGC | 36374 | 0.26 | No Hit |
| GGGTAGCCGACATCGCCGCCGACCCCGTGCGCTCGG | 34451 | 0.25 | No Hit |
| GGGGAAGAGAGGTGGCGACGACGCGGGGGACGACGG | 34332 | 0.25 | No Hit |
| CGGGTGGGTAGCCGACATCGCCGCCGACCCCGTGCG | 29912 | 0.22 | No Hit |
| GTCCGCGGGGCCCGACGCCGCGGGGGCGAAACCCGG | 27278 | 0.2 | No Hit |
| CGCGGCGTAGCGTCCGCGGGGCCCGACGCCGCGGGG | 24609 | 0.18 | No Hit |
| GTTCAACGTGACGGGCGAGAGGGCGGCCCCCTTTCC | 24440 | 0.18 | No Hit |
| CCAGGCTGGAGTGCAGTGGCTATTCACAGGCGCGAT | 24364 | 0.18 | No Hit |
| CCCGTGCGCTCGGCTTCGTCGGGAGACGCGTGACCG | 24293 | 0.18 | No Hit |
| ATCTGGCTTCCTCGGCCCCGGGATTCGGCGAAAGCT | 23706 | 0.17 | No Hit |
| GCCGACATCGCCGCCGACCCCGTGCGCTCGGCTTCG | 23380 | 0.17 | No Hit |
| CTCCGTTTCCGACCTGGGCCGGTTCACCCCTCCTT | 23247 | 0.17 | No Hit |
| CTCGCTATGTTGCCCAGGCTGGAGTGCAGTGGCTAT | 22703 | 0.17 | No Hit |
| GGAAGAAGGAGGGGGGGAAGAGAGGTGGCGACGACG | 22393 | 0.16 | No Hit |
| CCCTCCTTAGGCAACCTGGTGGTCCCCCGCTCCCGG | 21532 | 0.16 | No Hit |
| GGGAAGAGAGGTGGCGACGACGCGGGGGACGACGGG | 18485 | 0.13 | No Hit |
| CTCGTCGCGGCGTAGCGTCCGCGGGGCCCGACGCCG | 18300 | 0.13 | No Hit |
| CCCCTCCTTAGGCAACCTGGTGGTCCCCCGCTCCCG | 17137 | 0.12 | No Hit |
| CCTACTCGTCGCGGCGTAGCGTCCGCGGGGCCCGAC | 14854 | 0.11 | No Hit |
| CCAGAGTCGCCGCCGCCGCCGGCCCCCCCGAGTGTC | 14809 | 0.11 | No Hit |
| AGCCGACATCGCCGCCGACCCCGTGCGCTCGGCTTC | 14495 | 0.11 | No Hit |
| CCCAAGACGAACGGCTCTCCGCACCGGACCCCGGTC | 14119 | 0.1 | No Hit |
| AGCGTCCGCGGGGCCCGACGCCGCGGGGGCGAAACC | 14057 | 0.1 | No Hit |
| CCCCGAGTGTCCGGGCCCCCCGCCCCACCGGGGGCC | 13858 | 0.1 | No Hit |
| CTCGCGTCCAGAGTCGCCGCCGCCGCCGGCCCCCCC | 13777 | 0.1 | No Hit |

## Adapter Content

Produced by FastQC (version 0.13.0)
