# Supplementary material for: Bulk RNA sequencing dataset of hippocampi from RORγt transgenic mice and wild type littermates
Source: Data Brief. 2026 Oct 17;69:113361. doi: 10.1016/j.dib.2026.113361 (PMC13743875; doi:10.1016/j.dib.2026.113361)
Supplement: Supplementary file 2 [file mmc2.zip › Supplementary_Data_2_FastQC_MultiQC/fastqc/Tg2_TKS_001_05_R2_fastqc.html]

Tg2\_TKS\_001\_05\_R2.fastq.gz FastQC Report 

FastQC Report

�� 12 10�� 2026  
Tg2\_TKS\_001\_05\_R2.fastq.gz

## Summary

- Basic Statistics
- Per base sequence quality
- Per tile sequence quality
- Per sequence quality scores
- Per base sequence content
- Per sequence GC content
- Per base N content
- Sequence Length Distribution
- Sequence Duplication Levels
- Overrepresented sequences
- Adapter Content

## Basic Statistics

| Measure | Value |
| --- | --- |
| Filename | Tg2\_TKS\_001\_05\_R2.fastq.gz |
| File type | Conventional base calls |
| Encoding | Sanger / Illumina 1.9 |
| Total Sequences | 13754780 |
| Total Bases | 491.2 Mbp |
| Sequence length | 35-36 |
| Mean Length | 35 |
| Median Length | 36 |
| %GC | 57 |

## Per base sequence quality

## Per tile sequence quality

## Per sequence quality scores

## Per base sequence content

## Per sequence GC content

## Per base N content

## Sequence Length Distribution

## Sequence Duplication Levels

## Overrepresented sequences

| Sequence | Count | Percentage | Possible Source |
| --- | --- | --- | --- |
| CCTCTCTCGGGGCCCGGTGGGGGGCGGGGCGGACTG | 55468 | 0.4 | No Hit |
| CGGGACGGGAGCGGCCGCGGGTGCGCGTCTCTCGGG | 43513 | 0.32 | No Hit |
| CGCCTCTCTCGGGGCCCGGTGGGGGGCGGGGCGGAC | 41616 | 0.3 | No Hit |
| CGTCGTCCCCCGCGTCGTCGCCACCTCTCTTCCCCC | 39685 | 0.29 | No Hit |
| CTTCCACCGTGGCGGTGCGCCGCGACCGGCTCCGGG | 36306 | 0.26 | No Hit |
| CGGAACGGGACGGGAGCGGCCGCGGGTGCGCGTCTC | 35428 | 0.26 | No Hit |
| CGAGGCGCCGCCGCCCTCTCCCACGTCCGGGGAGAC | 24329 | 0.18 | No Hit |
| CTCCCACGTCCGGGGAGACCCCCCGTCCTTTCCGCC | 22372 | 0.16 | No Hit |
| CTCGGGGCCCGGTGGGGGGCGGGGCGGACTGTCCCC | 21205 | 0.15 | No Hit |
| CGGTGGCGCACGCCTGTAGTCCCAGCTACTCGGGN | 20916 | 0.15 | No Hit |
| GGACGAGGCGCCGCCGCCCTCTCCCACGTCCGGGG | 20624 | 0.15 | No Hit |
| CGTCCGTCCCCGCTCGGCGGGGTCCCCGCGTCGTCC | 20084 | 0.15 | No Hit |
| CCGCCCTCTCCCACGTCCGGGGAGACCCCCCGTCCT | 19967 | 0.15 | No Hit |
| CCCGGATAGCCGGGTCCCCGTCCGTCCCCGCTCGGC | 19274 | 0.14 | No Hit |
| CGGTGCGCCGCGACCGGCTCCGGGACGGCCGGGAN | 19105 | 0.14 | No Hit |
| CTGGACGAGGCGCCGCCGCCCTCTCCCACGTCCGGG | 18988 | 0.14 | No Hit |
| CCCACCCCCCTCCGTCGCCTCTCTCGGGGCCCGGTG | 18682 | 0.14 | No Hit |
| CCCAGCTACTCGGGAGGCTGAGACAGGAGGATCGCT | 18583 | 0.14 | No Hit |
| GGCGCCGCCGCCCTCTCCCACGTCCGGGGAGACCCC | 17336 | 0.13 | No Hit |
| CGGGAAGGCCCGGTGGGGAAGGTGGCTCGGGGGGGG | 17033 | 0.12 | No Hit |
| CCTCCGTCGCCTCTCTCGGGGCCCGGTGGGGGGCGG | 16926 | 0.12 | No Hit |
| CGGGGAGCCCGGCGGGTGCCGGCGCGGGTCCCCTCC | 16744 | 0.12 | No Hit |
| CTCTCTCGGGGCCCGGTGGGGGGCGGGGCGGACTGT | 15479 | 0.11 | No Hit |
| GGAGGATTCAACCCGGCGGCGCGCGTCCGGCCGTGC | 15316 | 0.11 | No Hit |
| CCGGCCGTCGCCGGCAGTCGGAACGGAACGGGACGG | 15285 | 0.11 | No Hit |
| CGGAACGGAACGGGACGGGAGCGGCCGCGGGTGCGC | 14992 | 0.11 | No Hit |
| CCGGCGGGTGCCGGCGCGGGTCCCCTCCCCGCGGGG | 14935 | 0.11 | No Hit |
| NNNNNNNNNNNNNNNNNNNNNNNNNNNNNNNNNNN | 14116 | 0.1 | No Hit |
| CGGCGGGTGCCGGCGCGGGTCCCCTCCCCGCGGGGC | 14088 | 0.1 | No Hit |
| CCCACGTCCGGGGAGACCCCCCGTCCTTTCCGCCCG | 13962 | 0.1 | No Hit |

## Adapter Content

Produced by FastQC (version 0.13.0)
