# Supplementary material for: Bulk RNA sequencing dataset of hippocampi from RORγt transgenic mice and wild type littermates
Source: Data Brief. 2026 Oct 17;69:113361. doi: 10.1016/j.dib.2026.113361 (PMC13743875; doi:10.1016/j.dib.2026.113361)
Supplement: Supplementary file 2 [file mmc2.zip › Supplementary_Data_2_FastQC_MultiQC/fastqc/Tg3_TKS_001_06_R1_fastqc.html]

Tg3\_TKS\_001\_06\_R1.fastq.gz FastQC Report 

FastQC Report

�� 12 10�� 2026  
Tg3\_TKS\_001\_06\_R1.fastq.gz

## Summary

- Basic Statistics
- Per base sequence quality
- Per tile sequence quality
- Per sequence quality scores
- Per base sequence content
- Per sequence GC content
- Per base N content
- Sequence Length Distribution
- Sequence Duplication Levels
- Overrepresented sequences
- Adapter Content

## Basic Statistics

| Measure | Value |
| --- | --- |
| Filename | Tg3\_TKS\_001\_06\_R1.fastq.gz |
| File type | Conventional base calls |
| Encoding | Sanger / Illumina 1.9 |
| Total Sequences | 13198555 |
| Total Bases | 471.3 Mbp |
| Sequence length | 35-36 |
| Mean Length | 35 |
| Median Length | 36 |
| %GC | 55 |

## Per base sequence quality

## Per tile sequence quality

## Per sequence quality scores

## Per base sequence content

## Per sequence GC content

## Per base N content

## Sequence Length Distribution

## Sequence Duplication Levels

## Overrepresented sequences

| Sequence | Count | Percentage | Possible Source |
| --- | --- | --- | --- |
| CTCGGCTTCGTCGGGAGACGCGTGACCGACGGTCCC | 77096 | 0.58 | No Hit |
| CGGGTATCTGGCTTCCTCGGCCCCGGGATTCGGCG | 59147 | 0.45 | No Hit |
| CTGGCTTCCTCGGCCCCGGGATTCGGCGAAAGCTGC | 49822 | 0.38 | No Hit |
| GGGGAAGAGAGGTGGCGACGACGCGGGGGACGACGG | 35974 | 0.27 | No Hit |
| CTGGAGTCTTGGAAGCTTGACTACCCTACGTTCTCC | 27649 | 0.21 | No Hit |
| GTCCGCGGGGCCCGACGCCGCGGGGGCGAAACCCGG | 23644 | 0.18 | No Hit |
| CCCGAGTGTCCGGGCCCCCCGCCCCACCGGGGGCCC | 23584 | 0.18 | No Hit |
| GGAAGAAGGAGGGGGGGAAGAGAGGTGGCGACGACG | 23057 | 0.17 | No Hit |
| GTCCAGAGTCGCCGCCGCCGCCGGCCCCCCCGAGTG | 21617 | 0.16 | No Hit |
| GTCGCGGCGTAGCGTCCGCGGGGCCCGACGCCGCGG | 21392 | 0.16 | No Hit |
| GGGAAGAGAGGTGGCGACGACGCGGGGGACGACGGG | 18731 | 0.14 | No Hit |
| NNNNNNNNNNNNNNNNNNNNNNNNNNNNNNNNNNN | 18043 | 0.14 | No Hit |
| CTCGCTATGTTGCCCAGGCTGGAGTGCAGTGGCTAT | 17375 | 0.13 | No Hit |
| GGGTAGCCGACATCGCCGCCGACCCCGTGCGCTCGG | 17304 | 0.13 | No Hit |
| ATCTGGCTTCCTCGGCCCCGGGATTCGGCGAAAGCT | 16921 | 0.13 | No Hit |
| CCAGGCTGGAGTGCAGTGGCTATTCACAGGCGCGAT | 16417 | 0.12 | No Hit |
| CGGGTGGGTAGCCGACATCGCCGCCGACCCCGTGCG | 15166 | 0.11 | No Hit |
| GCCGACATCGCCGCCGACCCCGTGCGCTCGGCTTCG | 15030 | 0.11 | No Hit |
| CCCGTGCGCTCGGCTTCGTCGGGAGACGCGTGACCG | 14927 | 0.11 | No Hit |
| CTCCGTTTCCGACCTGGGCCGGTTCACCCCTCCTT | 14676 | 0.11 | No Hit |
| CCCTCCTTAGGCAACCTGGTGGTCCCCCGCTCCCGG | 14480 | 0.11 | No Hit |
| CTCCTACTCGTCGCGGCGTAGCGTCCGCGGGGCCCG | 14337 | 0.11 | No Hit |
| CGCGGCGTAGCGTCCGCGGGGCCCGACGCCGCGGGG | 13208 | 0.1 | No Hit |

## Adapter Content

Produced by FastQC (version 0.13.0)
