# Supplementary material for: Bulk RNA sequencing dataset of hippocampi from RORγt transgenic mice and wild type littermates
Source: Data Brief. 2026 Oct 17;69:113361. doi: 10.1016/j.dib.2026.113361 (PMC13743875; doi:10.1016/j.dib.2026.113361)
Supplement: Supplementary file 2 [file mmc2.zip › Supplementary_Data_2_FastQC_MultiQC/fastqc/Tg3_TKS_001_06_R2_fastqc.html]

Tg3\_TKS\_001\_06\_R2.fastq.gz FastQC Report 

FastQC Report

�� 12 10�� 2026  
Tg3\_TKS\_001\_06\_R2.fastq.gz

## Summary

- Basic Statistics
- Per base sequence quality
- Per tile sequence quality
- Per sequence quality scores
- Per base sequence content
- Per sequence GC content
- Per base N content
- Sequence Length Distribution
- Sequence Duplication Levels
- Overrepresented sequences
- Adapter Content

## Basic Statistics

| Measure | Value |
| --- | --- |
| Filename | Tg3\_TKS\_001\_06\_R2.fastq.gz |
| File type | Conventional base calls |
| Encoding | Sanger / Illumina 1.9 |
| Total Sequences | 13198555 |
| Total Bases | 471 Mbp |
| Sequence length | 35-36 |
| Mean Length | 35 |
| Median Length | 36 |
| %GC | 55 |

## Per base sequence quality

## Per tile sequence quality

## Per sequence quality scores

## Per base sequence content

## Per sequence GC content

## Per base N content

## Sequence Length Distribution

## Sequence Duplication Levels

## Overrepresented sequences

| Sequence | Count | Percentage | Possible Source |
| --- | --- | --- | --- |
| CCTCTCTCGGGGCCCGGTGGGGGGCGGGGCGGACTG | 45006 | 0.34 | No Hit |
| CGCCTCTCTCGGGGCCCGGTGGGGGGCGGGGCGGAC | 33980 | 0.26 | No Hit |
| CGGGACGGGAGCGGCCGCGGGTGCGCGTCTCTCGGG | 28383 | 0.22 | No Hit |
| CTTCCACCGTGGCGGTGCGCCGCGACCGGCTCCGGG | 22517 | 0.17 | No Hit |
| CGTCGTCCCCCGCGTCGTCGCCACCTCTCTTCCCCC | 22435 | 0.17 | No Hit |
| CGGAACGGGACGGGAGCGGCCGCGGGTGCGCGTCTC | 22223 | 0.17 | No Hit |
| CGAGGCGCCGCCGCCCTCTCCCACGTCCGGGGAGAC | 20844 | 0.16 | No Hit |
| NNNNNNNNNNNNNNNNNNNNNNNNNNNNNNNNNNN | 20120 | 0.15 | No Hit |
| CTCCCACGTCCGGGGAGACCCCCCGTCCTTTCCGCC | 19218 | 0.15 | No Hit |
| CTCGGGGCCCGGTGGGGGGCGGGGCGGACTGTCCCC | 17413 | 0.13 | No Hit |
| GGACGAGGCGCCGCCGCCCTCTCCCACGTCCGGGG | 17287 | 0.13 | No Hit |
| CCGCCCTCTCCCACGTCCGGGGAGACCCCCCGTCCT | 16861 | 0.13 | No Hit |
| CTGGACGAGGCGCCGCCGCCCTCTCCCACGTCCGGG | 15751 | 0.12 | No Hit |
| GGCGCCGCCGCCCTCTCCCACGTCCGGGGAGACCCC | 15601 | 0.12 | No Hit |
| GGAGGATTCAACCCGGCGGCGCGCGTCCGGCCGTGC | 15399 | 0.12 | No Hit |
| CGTCCGTCCCCGCTCGGCGGGGTCCCCGCGTCGTCC | 14070 | 0.11 | No Hit |
| CGGTGCGCCGCGACCGGCTCCGGGACGGCCGGGAN | 13463 | 0.1 | No Hit |
| CGGGAAGGCCCGGTGGGGAAGGTGGCTCGGGGGGGG | 13214 | 0.1 | No Hit |

## Adapter Content

Produced by FastQC (version 0.13.0)
