# Supplementary material for: Bulk RNA sequencing dataset of hippocampi from RORγt transgenic mice and wild type littermates
Source: Data Brief. 2026 Oct 17;69:113361. doi: 10.1016/j.dib.2026.113361 (PMC13743875; doi:10.1016/j.dib.2026.113361)
Supplement: Supplementary file 2 [file mmc2.zip › Supplementary_Data_2_FastQC_MultiQC/fastqc/WT1_TKS_001_01_R1_fastqc.html]

WT1\_TKS\_001\_01\_R1.fastq.gz FastQC Report 

FastQC Report

�� 12 10�� 2026  
WT1\_TKS\_001\_01\_R1.fastq.gz

## Summary

- Basic Statistics
- Per base sequence quality
- Per tile sequence quality
- Per sequence quality scores
- Per base sequence content
- Per sequence GC content
- Per base N content
- Sequence Length Distribution
- Sequence Duplication Levels
- Overrepresented sequences
- Adapter Content

## Basic Statistics

| Measure | Value |
| --- | --- |
| Filename | WT1\_TKS\_001\_01\_R1.fastq.gz |
| File type | Conventional base calls |
| Encoding | Sanger / Illumina 1.9 |
| Total Sequences | 15232538 |
| Total Bases | 544.2 Mbp |
| Sequence length | 35-36 |
| Mean Length | 35 |
| Median Length | 36 |
| %GC | 58 |

## Per base sequence quality

## Per tile sequence quality

## Per sequence quality scores

## Per base sequence content

## Per sequence GC content

## Per base N content

## Sequence Length Distribution

## Sequence Duplication Levels

## Overrepresented sequences

| Sequence | Count | Percentage | Possible Source |
| --- | --- | --- | --- |
| CGGGTATCTGGCTTCCTCGGCCCCGGGATTCGGCG | 129787 | 0.85 | No Hit |
| CTGGCTTCCTCGGCCCCGGGATTCGGCGAAAGCTGC | 90735 | 0.6 | No Hit |
| GGGGAAGAGAGGTGGCGACGACGCGGGGGACGACGG | 79171 | 0.52 | No Hit |
| CTCGGCTTCGTCGGGAGACGCGTGACCGACGGTCCC | 78735 | 0.52 | No Hit |
| GGGTAGCCGACATCGCCGCCGACCCCGTGCGCTCGG | 47598 | 0.31 | No Hit |
| CCCGAGTGTCCGGGCCCCCCGCCCCACCGGGGGCCC | 44097 | 0.29 | No Hit |
| GGGAAGAGAGGTGGCGACGACGCGGGGGACGACGGG | 36698 | 0.24 | No Hit |
| GCCGACATCGCCGCCGACCCCGTGCGCTCGGCTTCG | 34499 | 0.23 | No Hit |
| GTCCGCGGGGCCCGACGCCGCGGGGGCGAAACCCGG | 34303 | 0.23 | No Hit |
| GTCCAGAGTCGCCGCCGCCGCCGGCCCCCCCGAGTG | 33708 | 0.22 | No Hit |
| CTGGAGTCTTGGAAGCTTGACTACCCTACGTTCTCC | 33577 | 0.22 | No Hit |
| ACGGGTATCTGGCTTCCTCGGCCCCGGGATTCGGCG | 33074 | 0.22 | No Hit |
| CGGGTGGGTAGCCGACATCGCCGCCGACCCCGTGCG | 32792 | 0.22 | No Hit |
| CCCGTGCGCTCGGCTTCGTCGGGAGACGCGTGACCG | 26604 | 0.17 | No Hit |
| GGCGACGACGCGGGGGACGACGGGGCCCCGCGGGG | 26338 | 0.17 | No Hit |
| GGAAGAAGGAGGGGGGGAAGAGAGGTGGCGACGACG | 25922 | 0.17 | No Hit |
| GTCGGCTGCTAGGCGCCGGCCGAGGCGAGGCGCCGC | 25117 | 0.16 | No Hit |
| GTCGCGGCGTAGCGTCCGCGGGGCCCGACGCCGCGG | 24931 | 0.16 | No Hit |
| CGGGCGAGAGGGCGGCCCCCTTTCCGGCCGCACCCC | 24620 | 0.16 | No Hit |
| CGCGGCGTAGCGTCCGCGGGGCCCGACGCCGCGGGG | 23459 | 0.15 | No Hit |
| NNNNNNNNNNNNNNNNNNNNNNNNNNNNNNNNNNN | 23247 | 0.15 | No Hit |
| GACGGGTATCTGGCTTCCTCGGCCCCGGGATTCGGC | 22199 | 0.15 | No Hit |
| ATCTGGCTTCCTCGGCCCCGGGATTCGGCGAAAGCT | 21756 | 0.14 | No Hit |
| GTTCAACGTGACGGGCGAGAGGGCGGCCCCCTTTCC | 21627 | 0.14 | No Hit |
| CGACGGGTATCTGGCTTCCTCGGCCCCGGGATTCGG | 21541 | 0.14 | No Hit |
| AGCGTCCGCGGGGCCCGACGCCGCGGGGGCGAAACC | 21276 | 0.14 | No Hit |
| AGGGGAACGACGCGCGGGTGGAGGGGTCGGGAGGA | 19116 | 0.13 | No Hit |
| CCAGGCTGGAGTGCAGTGGCTATTCACAGGCGCGAT | 18508 | 0.12 | No Hit |
| GTGGCGACGACGCGGGGGACGACGGGGCCCCGCGGG | 18067 | 0.12 | No Hit |
| CCCAAGACGAACGGCTCTCCGCACCGGACCCCGGTC | 17860 | 0.12 | No Hit |
| GTGGGTAGCCGACATCGCCGCCGACCCCGTGCGCTC | 17648 | 0.12 | No Hit |
| CGCGGCGACGGGTATCTGGCTTCCTCGGCCCCGGG | 16975 | 0.11 | No Hit |
| GGGAGGGGCGGCCCGGCCCCCACCCCCACGCCCGCC | 16298 | 0.11 | No Hit |
| GGGAAGAAGGAGGGGGGGAAGAGAGGTGGCGACGAC | 16129 | 0.11 | No Hit |
| GGAGGGGCGGCCCGGCCCCCACCCCCACGCCCGCCC | 15746 | 0.1 | No Hit |

## Adapter Content

Produced by FastQC (version 0.13.0)
