# Supplementary material for: Bulk RNA sequencing dataset of hippocampi from RORγt transgenic mice and wild type littermates
Source: Data Brief. 2026 Oct 17;69:113361. doi: 10.1016/j.dib.2026.113361 (PMC13743875; doi:10.1016/j.dib.2026.113361)
Supplement: Supplementary file 2 [file mmc2.zip › Supplementary_Data_2_FastQC_MultiQC/fastqc/WT1_TKS_001_01_R2_fastqc.html]

WT1\_TKS\_001\_01\_R2.fastq.gz FastQC Report 

FastQC Report

�� 12 10�� 2026  
WT1\_TKS\_001\_01\_R2.fastq.gz

## Summary

- Basic Statistics
- Per base sequence quality
- Per tile sequence quality
- Per sequence quality scores
- Per base sequence content
- Per sequence GC content
- Per base N content
- Sequence Length Distribution
- Sequence Duplication Levels
- Overrepresented sequences
- Adapter Content

## Basic Statistics

| Measure | Value |
| --- | --- |
| Filename | WT1\_TKS\_001\_01\_R2.fastq.gz |
| File type | Conventional base calls |
| Encoding | Sanger / Illumina 1.9 |
| Total Sequences | 15232538 |
| Total Bases | 544.1 Mbp |
| Sequence length | 35-36 |
| Mean Length | 35 |
| Median Length | 36 |
| %GC | 59 |

## Per base sequence quality

## Per tile sequence quality

## Per sequence quality scores

## Per base sequence content

## Per sequence GC content

## Per base N content

## Sequence Length Distribution

## Sequence Duplication Levels

## Overrepresented sequences

| Sequence | Count | Percentage | Possible Source |
| --- | --- | --- | --- |
| CCTCTCTCGGGGCCCGGTGGGGGGCGGGGCGGACTG | 92679 | 0.61 | No Hit |
| CGCCTCTCTCGGGGCCCGGTGGGGGGCGGGGCGGAC | 59780 | 0.39 | No Hit |
| CGTCGTCCCCCGCGTCGTCGCCACCTCTCTTCCCCC | 47240 | 0.31 | No Hit |
| CGAGGCGCCGCCGCCCTCTCCCACGTCCGGGGAGAC | 44740 | 0.29 | No Hit |
| CTTCCACCGTGGCGGTGCGCCGCGACCGGCTCCGGG | 40809 | 0.27 | No Hit |
| CGGGACGGGAGCGGCCGCGGGTGCGCGTCTCTCGGG | 40438 | 0.27 | No Hit |
| CGGTGCGCCGCGACCGGCTCCGGGACGGCCGGGAN | 39552 | 0.26 | No Hit |
| CGGGAAGGCCCGGTGGGGAAGGTGGCTCGGGGGGGG | 33882 | 0.22 | No Hit |
| CGTCCGTCCCCGCTCGGCGGGGTCCCCGCGTCGTCC | 32884 | 0.22 | No Hit |
| GGCGCCGCCGCCCTCTCCCACGTCCGGGGAGACCCC | 31170 | 0.2 | No Hit |
| GGACGAGGCGCCGCCGCCCTCTCCCACGTCCGGGG | 30695 | 0.2 | No Hit |
| CGGAACGGGACGGGAGCGGCCGCGGGTGCGCGTCTC | 29654 | 0.19 | No Hit |
| GGAGGATTCAACCCGGCGGCGCGCGTCCGGCCGTGC | 29515 | 0.19 | No Hit |
| CCGCCCTCTCCCACGTCCGGGGAGACCCCCCGTCCT | 28561 | 0.19 | No Hit |
| CTCGGGGCCCGGTGGGGGGCGGGGCGGACTGTCCCC | 27846 | 0.18 | No Hit |
| CTGGACGAGGCGCCGCCGCCCTCTCCCACGTCCGGG | 27727 | 0.18 | No Hit |
| CTCCCACGTCCGGGGAGACCCCCCGTCCTTTCCGCC | 26719 | 0.18 | No Hit |
| GCCGGGTCCCCGTCCGTCCCCGCTCGGCGGGGTCCC | 25588 | 0.17 | No Hit |
| NNNNNNNNNNNNNNNNNNNNNNNNNNNNNNNNNNN | 25449 | 0.17 | No Hit |
| CCCGGATAGCCGGGTCCCCGTCCGTCCCCGCTCGGC | 24417 | 0.16 | No Hit |
| CCCACCCCCCTCCGTCGCCTCTCTCGGGGCCCGGTG | 24370 | 0.16 | No Hit |
| CTCTCTCGGGGCCCGGTGGGGGGCGGGGCGGACTGT | 22223 | 0.15 | No Hit |
| CTCTCGGGGCCCGGTGGGGGGCGGGGCGGACTGTCC | 20015 | 0.13 | No Hit |
| CGCCGCCCTCTCCCACGTCCGGGGAGACCCCCCGTC | 19540 | 0.13 | No Hit |
| CCTCCGTCGCCTCTCTCGGGGCCCGGTGGGGGGCGG | 18724 | 0.12 | No Hit |
| CCGCGACCGGCTCCGGGACGGCCGGGAAGGCCCGGT | 17865 | 0.12 | No Hit |
| CGGGGCCCGGTGGGGGGCGGGGCGGACTGTCCCCN | 17164 | 0.11 | No Hit |
| CCCTCTCCCACGTCCGGGGAGACCCCCCGTCCTTTC | 16257 | 0.11 | No Hit |
| CGGGTCCCCGTCCGTCCCCGCTCGGCGGGGTCCCCG | 16242 | 0.11 | No Hit |
| CCCTCCGTCGCCTCTCTCGGGGCCCGGTGGGGGGCG | 15947 | 0.1 | No Hit |
| CGGTGGCGCACGCCTGTAGTCCCAGCTACTCGGGN | 15879 | 0.1 | No Hit |
| CGGCGGGTGCCGGCGCGGGTCCCCTCCCCGCGGGGC | 15779 | 0.1 | No Hit |
| CGGATAGCCGGGTCCCCGTCCGTCCCCGCTCGGCGG | 15402 | 0.1 | No Hit |

## Adapter Content

Produced by FastQC (version 0.13.0)
