# Supplementary material for: Bulk RNA sequencing dataset of hippocampi from RORγt transgenic mice and wild type littermates
Source: Data Brief. 2026 Oct 17;69:113361. doi: 10.1016/j.dib.2026.113361 (PMC13743875; doi:10.1016/j.dib.2026.113361)
Supplement: Supplementary file 2 [file mmc2.zip › Supplementary_Data_2_FastQC_MultiQC/fastqc/WT2_TKS_001_02_R1_fastqc.html]

WT2\_TKS\_001\_02\_R1.fastq.gz FastQC Report 

FastQC Report

�� 12 10�� 2026  
WT2\_TKS\_001\_02\_R1.fastq.gz

## Summary

- Basic Statistics
- Per base sequence quality
- Per tile sequence quality
- Per sequence quality scores
- Per base sequence content
- Per sequence GC content
- Per base N content
- Sequence Length Distribution
- Sequence Duplication Levels
- Overrepresented sequences
- Adapter Content

## Basic Statistics

| Measure | Value |
| --- | --- |
| Filename | WT2\_TKS\_001\_02\_R1.fastq.gz |
| File type | Conventional base calls |
| Encoding | Sanger / Illumina 1.9 |
| Total Sequences | 13453304 |
| Total Bases | 480.5 Mbp |
| Sequence length | 35-36 |
| Mean Length | 35 |
| Median Length | 36 |
| %GC | 56 |

## Per base sequence quality

## Per tile sequence quality

## Per sequence quality scores

## Per base sequence content

## Per sequence GC content

## Per base N content

## Sequence Length Distribution

## Sequence Duplication Levels

## Overrepresented sequences

| Sequence | Count | Percentage | Possible Source |
| --- | --- | --- | --- |
| CGGGTATCTGGCTTCCTCGGCCCCGGGATTCGGCG | 87177 | 0.65 | No Hit |
| CTGGCTTCCTCGGCCCCGGGATTCGGCGAAAGCTGC | 81402 | 0.61 | No Hit |
| CTCGGCTTCGTCGGGAGACGCGTGACCGACGGTCCC | 73102 | 0.54 | No Hit |
| GTCCAGAGTCGCCGCCGCCGCCGGCCCCCCCGAGTG | 64748 | 0.48 | No Hit |
| CCCGAGTGTCCGGGCCCCCCGCCCCACCGGGGGCCC | 49492 | 0.37 | No Hit |
| GTCGGCTGCTAGGCGCCGGCCGAGGCGAGGCGCCGC | 35205 | 0.26 | No Hit |
| GTTCAACGTGACGGGCGAGAGGGCGGCCCCCTTTCC | 33175 | 0.25 | No Hit |
| CTGGAGTCTTGGAAGCTTGACTACCCTACGTTCTCC | 32729 | 0.24 | No Hit |
| CCAGGCTGGAGTGCAGTGGCTATTCACAGGCGCGAT | 29209 | 0.22 | No Hit |
| GGGTAGCCGACATCGCCGCCGACCCCGTGCGCTCGG | 27168 | 0.2 | No Hit |
| CTCGCTATGTTGCCCAGGCTGGAGTGCAGTGGCTAT | 25672 | 0.19 | No Hit |
| CGGGTGGGTAGCCGACATCGCCGCCGACCCCGTGCG | 25345 | 0.19 | No Hit |
| GTCGCGGCGTAGCGTCCGCGGGGCCCGACGCCGCGG | 24068 | 0.18 | No Hit |
| CCAGAGTCGCCGCCGCCGCCGGCCCCCCCGAGTGTC | 23504 | 0.17 | No Hit |
| GCCGACATCGCCGCCGACCCCGTGCGCTCGGCTTCG | 23387 | 0.17 | No Hit |
| ATCTGGCTTCCTCGGCCCCGGGATTCGGCGAAAGCT | 23003 | 0.17 | No Hit |
| CCCGGAGCCGGTCGCGGCGCACCGCCACGGTGGAN | 22976 | 0.17 | No Hit |
| CTCCGTTTCCGACCTGGGCCGGTTCACCCCTCCTT | 21206 | 0.16 | No Hit |
| GTCCGCGGGGCCCGACGCCGCGGGGGCGAAACCCGG | 21094 | 0.16 | No Hit |
| CCCGTGCGCTCGGCTTCGTCGGGAGACGCGTGACCG | 19569 | 0.15 | No Hit |
| GGGGAAGAGAGGTGGCGACGACGCGGGGGACGACGG | 19434 | 0.14 | No Hit |
| CGCGGCGTAGCGTCCGCGGGGCCCGACGCCGCGGGG | 17516 | 0.13 | No Hit |
| CCCTCCTTAGGCAACCTGGTGGTCCCCCGCTCCCGG | 16837 | 0.13 | No Hit |
| CGGGCGAGAGGGCGGCCCCCTTTCCGGCCGCACCCC | 15906 | 0.12 | No Hit |
| CTCCTACTCGTCGCGGCGTAGCGTCCGCGGGGCCCG | 15416 | 0.11 | No Hit |
| GCCCAGGCTGGAGTGCAGTGGCTATTCACAGGCGCG | 13714 | 0.1 | No Hit |

## Adapter Content

Produced by FastQC (version 0.13.0)
