# Supplementary material for: Bulk RNA sequencing dataset of hippocampi from RORγt transgenic mice and wild type littermates
Source: Data Brief. 2026 Oct 17;69:113361. doi: 10.1016/j.dib.2026.113361 (PMC13743875; doi:10.1016/j.dib.2026.113361)
Supplement: Supplementary file 2 [file mmc2.zip › Supplementary_Data_2_FastQC_MultiQC/fastqc/WT3_TKS_001_03_R1_fastqc.html]

WT3\_TKS\_001\_03\_R1.fastq.gz FastQC Report 

FastQC Report

�� 12 10�� 2026  
WT3\_TKS\_001\_03\_R1.fastq.gz

## Summary

- Basic Statistics
- Per base sequence quality
- Per tile sequence quality
- Per sequence quality scores
- Per base sequence content
- Per sequence GC content
- Per base N content
- Sequence Length Distribution
- Sequence Duplication Levels
- Overrepresented sequences
- Adapter Content

## Basic Statistics

| Measure | Value |
| --- | --- |
| Filename | WT3\_TKS\_001\_03\_R1.fastq.gz |
| File type | Conventional base calls |
| Encoding | Sanger / Illumina 1.9 |
| Total Sequences | 14611817 |
| Total Bases | 521.9 Mbp |
| Sequence length | 35-36 |
| Mean Length | 35 |
| Median Length | 36 |
| %GC | 56 |

## Per base sequence quality

## Per tile sequence quality

## Per sequence quality scores

## Per base sequence content

## Per sequence GC content

## Per base N content

## Sequence Length Distribution

## Sequence Duplication Levels

## Overrepresented sequences

| Sequence | Count | Percentage | Possible Source |
| --- | --- | --- | --- |
| CTCGGCTTCGTCGGGAGACGCGTGACCGACGGTCCC | 112929 | 0.77 | No Hit |
| CGGGTATCTGGCTTCCTCGGCCCCGGGATTCGGCG | 88520 | 0.61 | No Hit |
| CTGGCTTCCTCGGCCCCGGGATTCGGCGAAAGCTGC | 74577 | 0.51 | No Hit |
| GTCCAGAGTCGCCGCCGCCGCCGGCCCCCCCGAGTG | 43894 | 0.3 | No Hit |
| GGGGAAGAGAGGTGGCGACGACGCGGGGGACGACGG | 41900 | 0.29 | No Hit |
| CCCGAGTGTCCGGGCCCCCCGCCCCACCGGGGGCCC | 41336 | 0.28 | No Hit |
| GTCCGCGGGGCCCGACGCCGCGGGGGCGAAACCCGG | 34397 | 0.24 | No Hit |
| CTGGAGTCTTGGAAGCTTGACTACCCTACGTTCTCC | 32577 | 0.22 | No Hit |
| GTCGCGGCGTAGCGTCCGCGGGGCCCGACGCCGCGG | 31870 | 0.22 | No Hit |
| GGGTAGCCGACATCGCCGCCGACCCCGTGCGCTCGG | 28583 | 0.2 | No Hit |
| CCAGGCTGGAGTGCAGTGGCTATTCACAGGCGCGAT | 26440 | 0.18 | No Hit |
| CTCGCTATGTTGCCCAGGCTGGAGTGCAGTGGCTAT | 26436 | 0.18 | No Hit |
| GCCGACATCGCCGCCGACCCCGTGCGCTCGGCTTCG | 25916 | 0.18 | No Hit |
| GTCGGCTGCTAGGCGCCGGCCGAGGCGAGGCGCCGC | 25911 | 0.18 | No Hit |
| GTTCAACGTGACGGGCGAGAGGGCGGCCCCCTTTCC | 25685 | 0.18 | No Hit |
| CGGGTGGGTAGCCGACATCGCCGCCGACCCCGTGCG | 25489 | 0.17 | No Hit |
| CCCGTGCGCTCGGCTTCGTCGGGAGACGCGTGACCG | 24985 | 0.17 | No Hit |
| GGGAAGAGAGGTGGCGACGACGCGGGGGACGACGGG | 23185 | 0.16 | No Hit |
| ATCTGGCTTCCTCGGCCCCGGGATTCGGCGAAAGCT | 22686 | 0.16 | No Hit |
| CTCCGTTTCCGACCTGGGCCGGTTCACCCCTCCTT | 20750 | 0.14 | No Hit |
| CGCGGCGTAGCGTCCGCGGGGCCCGACGCCGCGGGG | 20516 | 0.14 | No Hit |
| GGAAGAAGGAGGGGGGGAAGAGAGGTGGCGACGACG | 19689 | 0.13 | No Hit |
| AGCGTCCGCGGGGCCCGACGCCGCGGGGGCGAAACC | 18679 | 0.13 | No Hit |
| CCCTCCTTAGGCAACCTGGTGGTCCCCCGCTCCCGG | 18290 | 0.13 | No Hit |
| CCCAAGACGAACGGCTCTCCGCACCGGACCCCGGTC | 18124 | 0.12 | No Hit |
| CTCCTACTCGTCGCGGCGTAGCGTCCGCGGGGCCCG | 17161 | 0.12 | No Hit |
| NNNNNNNNNNNNNNNNNNNNNNNNNNNNNNNNNNN | 16089 | 0.11 | No Hit |
| CCAGAGTCGCCGCCGCCGCCGGCCCCCCCGAGTGTC | 15236 | 0.1 | No Hit |

## Adapter Content

Produced by FastQC (version 0.13.0)
