# Supplementary material for: Bulk RNA sequencing dataset of hippocampi from RORγt transgenic mice and wild type littermates
Source: Data Brief. 2026 Oct 17;69:113361. doi: 10.1016/j.dib.2026.113361 (PMC13743875; doi:10.1016/j.dib.2026.113361)
Supplement: Supplementary file 2 [file mmc2.zip › Supplementary_Data_2_FastQC_MultiQC/fastqc/WT3_TKS_001_03_R2_fastqc.html]

WT3\_TKS\_001\_03\_R2.fastq.gz FastQC Report 

FastQC Report

�� 12 10�� 2026  
WT3\_TKS\_001\_03\_R2.fastq.gz

## Summary

- Basic Statistics
- Per base sequence quality
- Per tile sequence quality
- Per sequence quality scores
- Per base sequence content
- Per sequence GC content
- Per base N content
- Sequence Length Distribution
- Sequence Duplication Levels
- Overrepresented sequences
- Adapter Content

## Basic Statistics

| Measure | Value |
| --- | --- |
| Filename | WT3\_TKS\_001\_03\_R2.fastq.gz |
| File type | Conventional base calls |
| Encoding | Sanger / Illumina 1.9 |
| Total Sequences | 14611817 |
| Total Bases | 521.7 Mbp |
| Sequence length | 35-36 |
| Mean Length | 35 |
| Median Length | 36 |
| %GC | 56 |

## Per base sequence quality

## Per tile sequence quality

## Per sequence quality scores

## Per base sequence content

## Per sequence GC content

## Per base N content

## Sequence Length Distribution

## Sequence Duplication Levels

## Overrepresented sequences

| Sequence | Count | Percentage | Possible Source |
| --- | --- | --- | --- |
| CCTCTCTCGGGGCCCGGTGGGGGGCGGGGCGGACTG | 63380 | 0.43 | No Hit |
| CGCCTCTCTCGGGGCCCGGTGGGGGGCGGGGCGGAC | 49443 | 0.34 | No Hit |
| CGGGACGGGAGCGGCCGCGGGTGCGCGTCTCTCGGG | 40038 | 0.27 | No Hit |
| CGTCGTCCCCCGCGTCGTCGCCACCTCTCTTCCCCC | 34822 | 0.24 | No Hit |
| CTTCCACCGTGGCGGTGCGCCGCGACCGGCTCCGGG | 31237 | 0.21 | No Hit |
| CGGAACGGGACGGGAGCGGCCGCGGGTGCGCGTCTC | 28362 | 0.19 | No Hit |
| CTCGGGGCCCGGTGGGGGGCGGGGCGGACTGTCCCC | 26333 | 0.18 | No Hit |
| CGAGGCGCCGCCGCCCTCTCCCACGTCCGGGGAGAC | 25889 | 0.18 | No Hit |
| CTCCCACGTCCGGGGAGACCCCCCGTCCTTTCCGCC | 24497 | 0.17 | No Hit |
| CGTCCGTCCCCGCTCGGCGGGGTCCCCGCGTCGTCC | 24102 | 0.16 | No Hit |
| CCGCCCTCTCCCACGTCCGGGGAGACCCCCCGTCCT | 22760 | 0.16 | No Hit |
| CCCGGATAGCCGGGTCCCCGTCCGTCCCCGCTCGGC | 21213 | 0.15 | No Hit |
| GGACGAGGCGCCGCCGCCCTCTCCCACGTCCGGGG | 21153 | 0.14 | No Hit |
| GGAGGATTCAACCCGGCGGCGCGCGTCCGGCCGTGC | 20992 | 0.14 | No Hit |
| CGGGAAGGCCCGGTGGGGAAGGTGGCTCGGGGGGGG | 20860 | 0.14 | No Hit |
| CGGTGCGCCGCGACCGGCTCCGGGACGGCCGGGAN | 20442 | 0.14 | No Hit |
| GGCGCCGCCGCCCTCTCCCACGTCCGGGGAGACCCC | 20078 | 0.14 | No Hit |
| CTGGACGAGGCGCCGCCGCCCTCTCCCACGTCCGGG | 18423 | 0.13 | No Hit |
| NNNNNNNNNNNNNNNNNNNNNNNNNNNNNNNNNNN | 18235 | 0.12 | No Hit |
| CTCTCTCGGGGCCCGGTGGGGGGCGGGGCGGACTGT | 17868 | 0.12 | No Hit |
| CGGTGGCGCACGCCTGTAGTCCCAGCTACTCGGGN | 17799 | 0.12 | No Hit |
| CCTCCGTCGCCTCTCTCGGGGCCCGGTGGGGGGCGG | 17545 | 0.12 | No Hit |
| CCCACCCCCCTCCGTCGCCTCTCTCGGGGCCCGGTG | 17359 | 0.12 | No Hit |
| GCCGGGTCCCCGTCCGTCCCCGCTCGGCGGGGTCCC | 16505 | 0.11 | No Hit |
| CGGGTCCCCGTCCGTCCCCGCTCGGCGGGGTCCCCG | 15595 | 0.11 | No Hit |
| CGCCGCCCTCTCCCACGTCCGGGGAGACCCCCCGTC | 15540 | 0.11 | No Hit |
| CCCAGCTACTCGGGAGGCTGAGACAGGAGGATCGCT | 15112 | 0.1 | No Hit |
| CGGATAGCCGGGTCCCCGTCCGTCCCCGCTCGGCGG | 14672 | 0.1 | No Hit |

## Adapter Content

Produced by FastQC (version 0.13.0)
